# Unraveling the Physiological Roles of the Cyanobacterium *Geitlerinema* sp. BBD and Other Black Band Disease Community Members through Genomic Analysis of a Mixed Culture

**DOI:** 10.1371/journal.pone.0157953

**Published:** 2016-06-23

**Authors:** Paul A. Den Uyl, Laurie L. Richardson, Sunit Jain, Gregory J. Dick

**Affiliations:** 1 Department of Earth and Environmental Sciences, University of Michigan, Ann Arbor, MI, 48109–1005, United States of America; 2 Ecology and Evolutionary Biology, University of Michigan, Ann Arbor, MI, 48109–1048, United States of America; 3 Center for Computational Medicine and Bioinformatics, University of Michigan, Ann Arbor, MI, 48109–2218, United States of America; 4 Department of Biological Sciences, Florida International University, Miami, FL 33199, United States of America; U.S. Geological Survey, UNITED STATES

## Abstract

Black band disease (BBD) is a cyanobacterial-dominated polymicrobial mat that propagates on and migrates across coral surfaces, necrotizing coral tissue. Culture-based laboratory studies have investigated cyanobacteria and heterotrophic bacteria isolated from BBD, but the metabolic potential of various BBD microbial community members and interactions between them remain poorly understood. Here we report genomic insights into the physiological and metabolic potential of the BBD-associated cyanobacterium *Geitlerinema* sp. BBD 1991 and six associated bacteria that were also present in the non-axenic culture. The essentially complete genome of *Geitlerinema* sp. BBD 1991 contains a sulfide quinone oxidoreductase gene for oxidation of sulfide, suggesting a mechanism for tolerating the sulfidic conditions of BBD mats. Although the operon for biosynthesis of the cyanotoxin microcystin was surprisingly absent, potential relics were identified. Genomic evidence for mixed-acid fermentation indicates a strategy for energy metabolism under the anaerobic conditions present in BBD during darkness. Fermentation products may supply carbon to BBD heterotrophic bacteria. Among the six associated bacteria in the culture, two are closely related to organisms found in culture-independent studies of diseased corals. Their metabolic pathways for carbon and sulfur cycling, energy metabolism, and mechanisms for resisting coral defenses suggest adaptations to the coral surface environment and biogeochemical roles within the BBD mat. Polysulfide reductases were identified in a Flammeovirgaceae genome (Bacteroidetes) and the *sox* pathway for sulfur oxidation was found in the genome of a Rhodospirillales bacterium (Alphaproteobacteria), revealing mechanisms for sulfur cycling, which influences virulence of BBD. Each genomic bin possessed a pathway for conserving energy from glycerol degradation, reflecting adaptations to the glycerol-rich coral environment. The presence of genes for detoxification of reactive oxygen species and resistance to antibiotics suggest mechanisms for combating coral defense strategies. This study builds upon previous research on BBD and provides new insights into BBD disease etiology.

## Introduction

Coral reefs are a diverse and resource-rich habitat, teeming with biological activity that supports an estimated 25% of all marine life. Yet it is well known that coral reefs are degrading on a worldwide basis. In particular, coral diseases, including black band disease (BBD), are recognized as a significant threat to coral reefs and associated ecosystem services [1, 2].

BBD is characterized by a dark cyanobacterial-dominated mat that is present as a band that migrates across the surface of infected corals [3]. It is similar to cyanobacterial mats found in other environments, such as hot spring outflows and hypersaline benthic zones, in that it contains strong vertical microgradients of oxygen and sulfide within the 1 mm thick band [4]. Studies using oxygen and sulfide sensitive microelectrodes have revealed that the band is fully anoxic and sulfide-rich at night, while during the day the surface of the band is supersaturated with oxygen. The oxygen/sulfide interface migrates vertically within the mat throughout the day/night cycle [4]. The band, which is dark red due to the cyanobacterial pigment phycoerythrin and appears black *in situ*, is known to consist of a pathogenic, polymicrobial community that includes, in addition to photosynthetic cyanobacteria, large populations of sulfate reducing bacteria, sulfide oxidizing bacteria, and numerous bacterial heterotrophs [5, 6]. Physiological processes of these BBD community members create and maintain the dynamic BBD chemical microenvironment, which is an important factor in pathogenicity [6, 7, 8, 9]. As the band moves across a coral colony, typically at rates of approximately 3 mm/day, but up to 1 cm/ day, it completely lyses coral tissue, leaving behind exposed coral skeleton. Coral tissue lysis is the result of exposure to the band itself, which is anoxic at the base and contains at least two toxins, sulfide and microcystin. The combination of anoxia and these toxins has been experimentally shown to lyse living coral tissue [10, 11]. For many BBD-susceptible corals, particularly scleractinian corals which grow on the order of 1 cm in circumference per year, the rate of tissue lysis can cause complete mortality of entire coral colonies and thus impact the biological and geological structure of coral reef environments [12, 13].

The dominance of the BBD microbial mat by gliding, filamentous, non-heterocystous cyanobacteria has been recognized since the first observations of BBD by Antonius in the 1970s [14, 15]. It is now known that, on a worldwide basis, there is one dominant BBD cyanobacterial ribotype, recently classified as the new genus and species *Roseofilum reptotaenium* [16, 17]. This ribotype has been found in BBD throughout the Caribbean [18], as well as on the Great Barrier Reef [19] and in the Red Sea [20]. Unlike BBD in the Indo-Pacific and the Red Sea, BBD in the Caribbean has been shown to contain several additional genera of cyanobacteria [21, 18], all of which have been shown to produce the cyanotoxin microcystin [22, 23, 24, 25]. In addition to toxin production, BBD cyanobacterial filaments play an important role in BBD by forming the physical matrix of the mat community [11].

The BBD microbial mat also contains a diversity of non-photosynthetic bacteria, some of which have been proposed as BBD pathogens [26]. These include sulfide-oxidizing bacteria, sulfate-reducing bacteria, and other heterotrophic bacteria [26, 27, 28, 29, 30]. The sulfide produced in the mat has been attributed to dissimilatory sulfate-reducing bacteria, primarily of the genus *Desulfovibrio* [27, 31]. Sulfide-oxidizing bacteria associated with BBD include *Beggiatoa* [32] and others [33], and may potentially play a role in detoxification of sulfide. A wide variety of BBD heterotrophic bacteria have been detected in BBD, but little is known about them beyond their taxonomy. The composition of this community was recently characterized through a meta-analysis of 87 published 16S rRNA gene clone libraries produced from individual samples of BBD [33].

Cyanobacteria are an important component of the pathogenesis of BBD. While some BBD cyanobacteria have been isolated into culture in the laboratory, none are axenic, and it may be that they require associated bacteria to thrive (discussed in [16]). However, laboratory experiments with non-axenic cultures have provided much useful information about the ecological physiology of BBD cyanobacteria and their relationship to BBD etiology. One such culture, designated *Geitlerinema* BBD 1991, was derived from a single BBD cyanobacterial filament and consists of the cyanobacterium *Geitlerinema* sp. BBD 1991 and several other bacteria presumably sourced from BBD [34]. This mixed culture has been extensively studied, and has provided insights into BBD cyanobacterial primary and secondary metabolism as well as how these may influence the functioning of the BBD community and its pathology. Consistent with its sulfide-rich BBD mat habitat, *Geitlerinema* sp. BBD 1991 is resistant to sulfide and able to perform sulfide-resistant oxygenic photosynthesis. However, it is unable to conduct anoxygenic photosynthesis using sulfide as an electron donor [35, 36]. *Geitlerinema* sp. BBD 1991 can survive under dark, anoxic conditions, presumably via fermentation [34], and has been shown to produce microcystin [23]. Antibacterials are also produced in the culture and are thought to take part in structuring the BBD microbial community [37] as well as functioning as additional virulence factors.

In this study, we conducted metagenomic sequencing of the non-axenic laboratory culture of *Geitlerinema* sp. BBD 1991. Through genomic assembly and binning, we reconstructed essentially complete genomes from the cyanobacterium (*Geitlerinema*) as well as six co-cultured bacteria. These genomes revealed the physiological and metabolic potential of the cultured members, providing insights into their adaptations to the BBD environment, potential interactions between community members, and contributions to disease pathogenicity. Although the enrichment culture is not a perfect representation of the BBD community, these genomic insights provide a foundation for analyzing BBD metagenomic datasets and suggest new hypotheses to test experimentally.

## Materials and Methods

### Culturing and maintenance

The laboratory enrichment culture was sourced from a single cyanobacterial filament obtained from a fresh BBD sample collected on Algae Reef (N 25' 08.799 W 80' 17.579). At the time of collection (1991) this area was within the Key Largo National Marine Sanctuary, and the collection was permitted by that organization. Early studies referred to this culture as *Phormidium corallyticum* due to morphology and based on the literature (in particular [3]), but phylogenetic studies showed it to be a member of the genus *Geitlerinema* [22]. Isolation and maintenance of the culture was carried out using ASNIII mineral media. For maintenance, 125 ml Erlenmeyer flasks were used with 25 ml of ASNIII agar solution initially added and allowed to solidify. 25 ml of additional ASNIII solution without agar was then added to the flask before culture transfer. Cultures were incubated in natural sunlight at room temperature (20–25°C). Henceforth, we refer to the mixed culture as *Geitlerinema* BBD 1991 and to the cyanobacterial species within the mixed culture as *Geitlerinema* sp. BBD 1991.

### DNA extraction, genome sequencing, and assembly

DNA was extracted from 0.5 grams of biomass from the *Geitlerinema* BBD 1991 culture using the Fast DNA spin kit for soil (MP Biomedicals, Solon, OH, USA) and Fastprep-24 Bead Beater (MP Biomedicals, Solon, OH, USA) in accordance with manufacturer directions, except that 0.3 grams of beads were used for bead beating. DNA was quantified using the Quant-IT PicoGreen dsDNA reagent and kit (Invitrogen, Grand Island, NY, USA) and submitted to the University of Michigan DNA Sequencing Core for one lane of Illumina HiSeq PE100 sequencing.

Genomic reads were dereplicated (100% identity over 100% lengths), then trimmed with the adaptive read trimmer, Sickle. Whole genome *de novo assemblies* were performed using IDBA-UD [38] with the following parameters:—mink 50,—maxk 92,—step 4 or 6,—min_contig 500. Binning of assembled scaffolds was performed using tetranucleotide frequencies in emergent self-organizing maps (ESOM) [39] with a contig length minimum cutoff of 2.5kb and window size of 5 kb. Reference genomes used in the ESOM were as follows (NCBI accession numbers in parentheses): *Arthrospira platensis* C1 (NZ_CM001632), *Geitlerinema* sp. PCC 7407 (NC_019703), *Microcoleus chthonoplastes* PCC7424 (NZ_DS989841.1), *Oscillatoria* sp. PCC 6506 (NZ_CACA01000001), *Prochlorococcus marinus* str. MIT 9211 (NC_009976), *Synechococcus elongatus* PCC 7942 (NC_007595), and *Trichodesmium erythraeum* IMS101 (NC_008312).

### Annotation, analysis methods, and completeness

Assembled scaffolds were annotated for genes and pathways using the Integrated Microbial Genomes Expert Review (IMG-ER) automated pipeline from the Joint Genomes Institute (JGI), Pathway Tools from BioCyc [40], and AntiSMASH [41]. Unless noted, all genes and pathways discussed underwent a TBLASTN analyses with a 60% sequence identity and 1e^-5^ e-value cutoff to their respective protein query downloaded from NCBI. The TBLASTN to search cyanobacterial genomes on the JGI Integrated Microbial Genomes website was conducted in November of 2014, when there were 97 cyanobacterial genomes available. Genome completeness of each genomic bin was assessed by searching for 35 universally conserved housekeeping genes [42].

16S rRNA gene sequences were identified by BLASTN of contigs against the SILVA Small Subunit rRNA database version 111 [43, 44]. Taxonomic classification was conducted with PhyloSift [45]. For bins containing 16S sequences, a maximum-likelihood tree was created with RAxML [46]. Sequences were aligned using mothur [47] with the GreenGenes database and bootstrapped 5000 times. For genomic bins without assembled 16S rRNA sequences, trees were made with amino acid sequences of ribosomal protein S3, again using RAxML. Amino acid trees were aligned using MUSCLE with default parameters and bootstrapped 1000 times.

## Results and Discussion

### Assembly and identification of genomic bins

**Fig 1:**
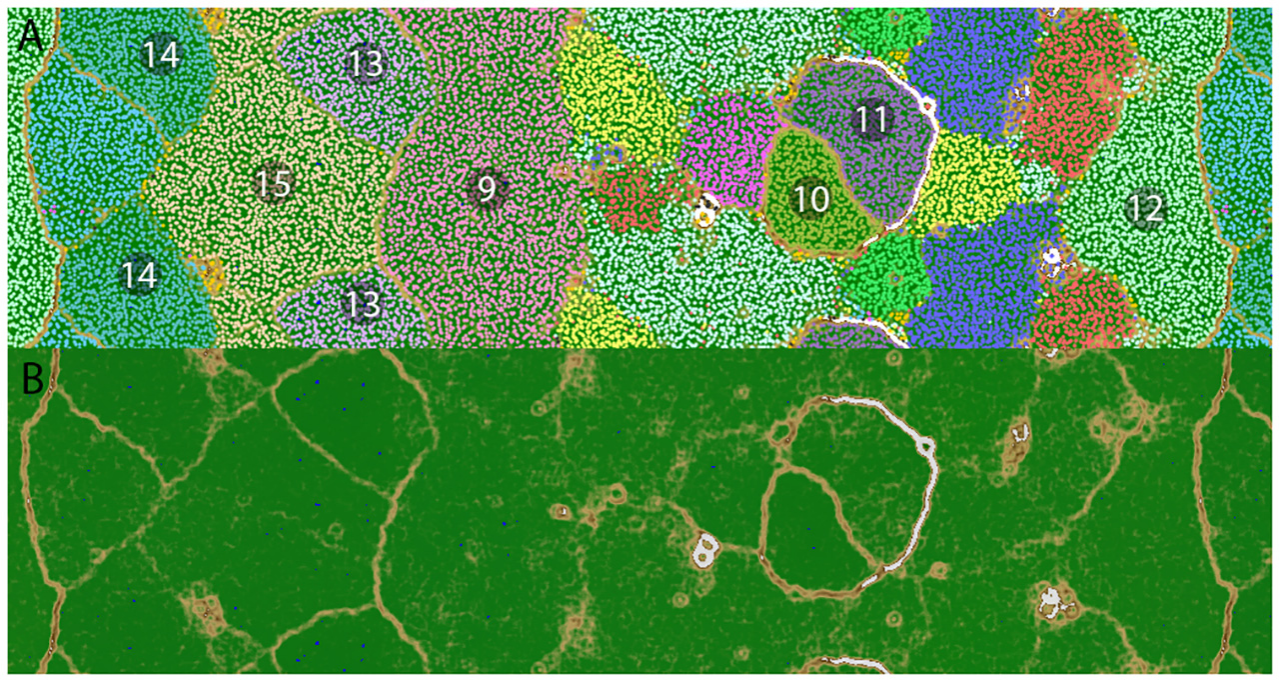
ESOM binning of the *Geitlerinema* BBD 1991 culture and reference genome bins. ESOM was used to assign sequence fragments to particular genomic bins on the basis of tetranucleotide frequency. The map is continuous from both top to bottom and side to side. Regions are numbered and highlighted in order to label each respective bin: (9) *Geitlerinema* sp. BBD 1991, pink; (10) *Oceanicaulis*, greenish-yellow; (11) *Planctomycetaceae*, purple; (12) *Flammeovirgaceae*, light green; (13) *Parvularcula*, lavender; (14) *Novispirillum*, teal; and (15) *Thioalkalivibrio*, yellow. Reference bins are colored and labeled as follows: *Arthrospira platensis* C1, reddish-orange; *Geitlerinema* sp. PCC 7407, blue; *Microcoleus chthonoplastes* PCC7424, light green; *Oscillatoria* sp. PCC 6506, yellow; *Prochlorococcus marinus* str. MIT 9211, green; *Synechococcus elongatus* PCC 7942, pink; and *Trichodesmium erythraeum* IMS101, purple. Panel (**A**) shows data points representing each 5-kb sequence window and **(B)** shows the same map with data points removed, revealing the topographic representation of the structure of the underlying tetranucleotide frequency data. Colors represent the differences in tetranucleotide frequency profiles between nodes of the ESOM matrix, with high 'elevations' (brown, white) indicating large differences in tetranucleotide frequency between points (and thus representing divisions between genomic bins) and green and blue indicating small differences in tetranucleotide frequency between points (expected within individual bins).

Genome assembly and binning by tetranucleotide frequency with ESOM revealed seven genomic bins from the *Geitlerinema* BBD 1991 culture (Fig 1). In addition to *Geitlerinema* sp., bins are apparent from three alphaproteobacteria, one gammaproteobacterium, one planctomycetes, and one bacteroidetes. Each bin possesses all 35 universal housekeeping genes in at least one copy. Bin 15 possesses two complete sets of the 35 housekeeping genes, implying the presence of two distinct genotypes. Taken together with coverage, which ranged from 32-167x, these results suggest that the genome bins produced here represent essentially complete genomes (Table 1).

**Table 1:** Summary of genomic bins. Genus level threshold is defined at 94.5% per Yarza et al. (2014) [44].

	Consensus Identity	16S (% ID)	PhyloSift Taxonomy (% of Bin)	# of Scaff.	Total Length (Mb)	% GC	AvgCov
Bin 9	Cyanobacteria;Oscillatoriophycideae;Oscillatoriales;*Geitlerinema*;*Geitlerinema* sp. BBD 1991	*Geitlerinema* sp. BBD (EF372580) (99%)	*Geitlerinema* (53%)Cyanobacteria (100%)	113	6.0	50	68
Bin 10	Proteobacteria;Alphaproteobacteria;Rhodobacterales;Hyphomonadaceae;*Oceanicaulis*	N/A	*Oceanicaulis* (79%)Proteobacteria (91%)	89	3.2	65	39
Bin 11	Planctomycetes;Planctomycetia;Planctomycetales;Planctomycetaceae	*Planctomycete* GMD21C08 (AY162119) (92%)	Planctomycetaceae (8%)Planctomycetes (27%)	405	4.9	68	59
Bin 12	Bacteroidetes;Cytophagia;Cytophagales;Flammeovirgaceae	*Flexibacter aggregans* IFO 15974 (AB078038) (94%)	Flammeovirgaceae (85%)Bacteroidetes (100%)	608	8.6	45	32
Bin 13	Proteobacteria;Alphaproteobacteria;Parvularculales;Parvularculaceae;*Parvularcula*	*Parvularcula* sp. P33 (EU851414) (96%)	*Parvularcula* (16%)Proteobacteria (87%)	36	4.2	65	167
Bin 14	Proteobacteria;Alphaproteobacteria;Rhodospirillales	N/A	Rhodospirillales (19%)Proteobacteria (74%)	21	5.5	66	116
Bin 15	Proteobacteria;Gammaproteobacteria;Chromatiales;Ectothiorhodospiraceae	N/A	Ectothiorhodospiraceae (22%)Proteobacteria (87%)	128	10.9	62	107

**Fig 2:**
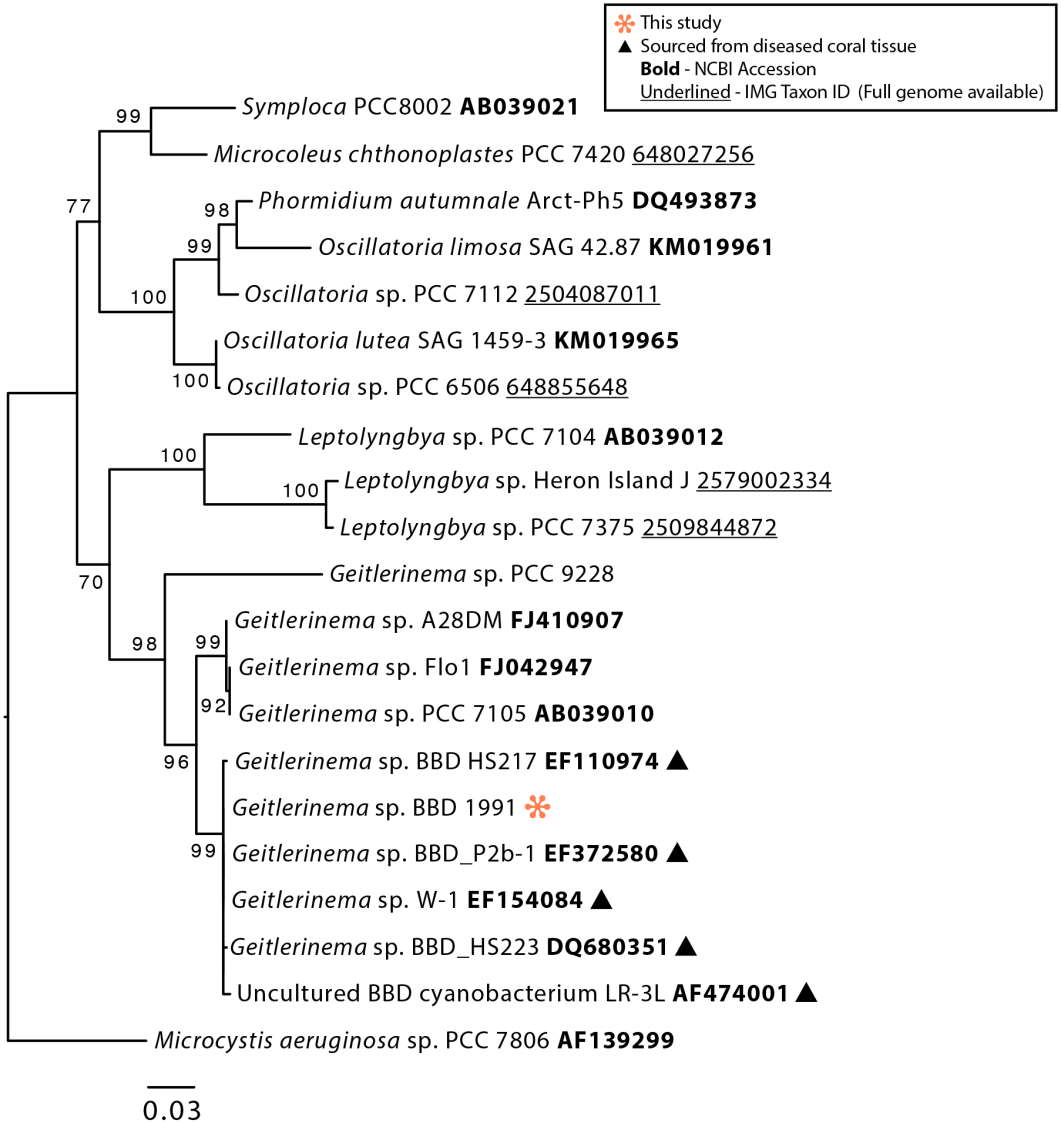
Phylogenetic tree of the 16S rRNA gene of *Geitlerinema* sp. BBD 1991 and selected members of the *Oscillatoriales*, Cyanobacteria. Bootstrap values are the result of 5000 iterations.

Phylogenetic analysis of the 16S rRNA gene from *Geitlerinema* sp. BBD 1991 showed that it clusters tightly with other strains isolated from BBD (Fig 2). It is also closely related to sp. PCC 7105, for which genome sequencing was recently conducted as part of the CyanoGEBA project [48]. Consistent with the genome of *Geitlerinema* sp. PCC 7105, which has an exceptional array of clustered regularly interspaced short palindromic repeats (CRISPRs) [49, 48], the *Geitlerinema* sp. BBD 1991 genome contains at least 12 CRISPR loci and six *cas* genes, suggesting that it faces viral predation pressure (S1 Table).

**Fig 3:**
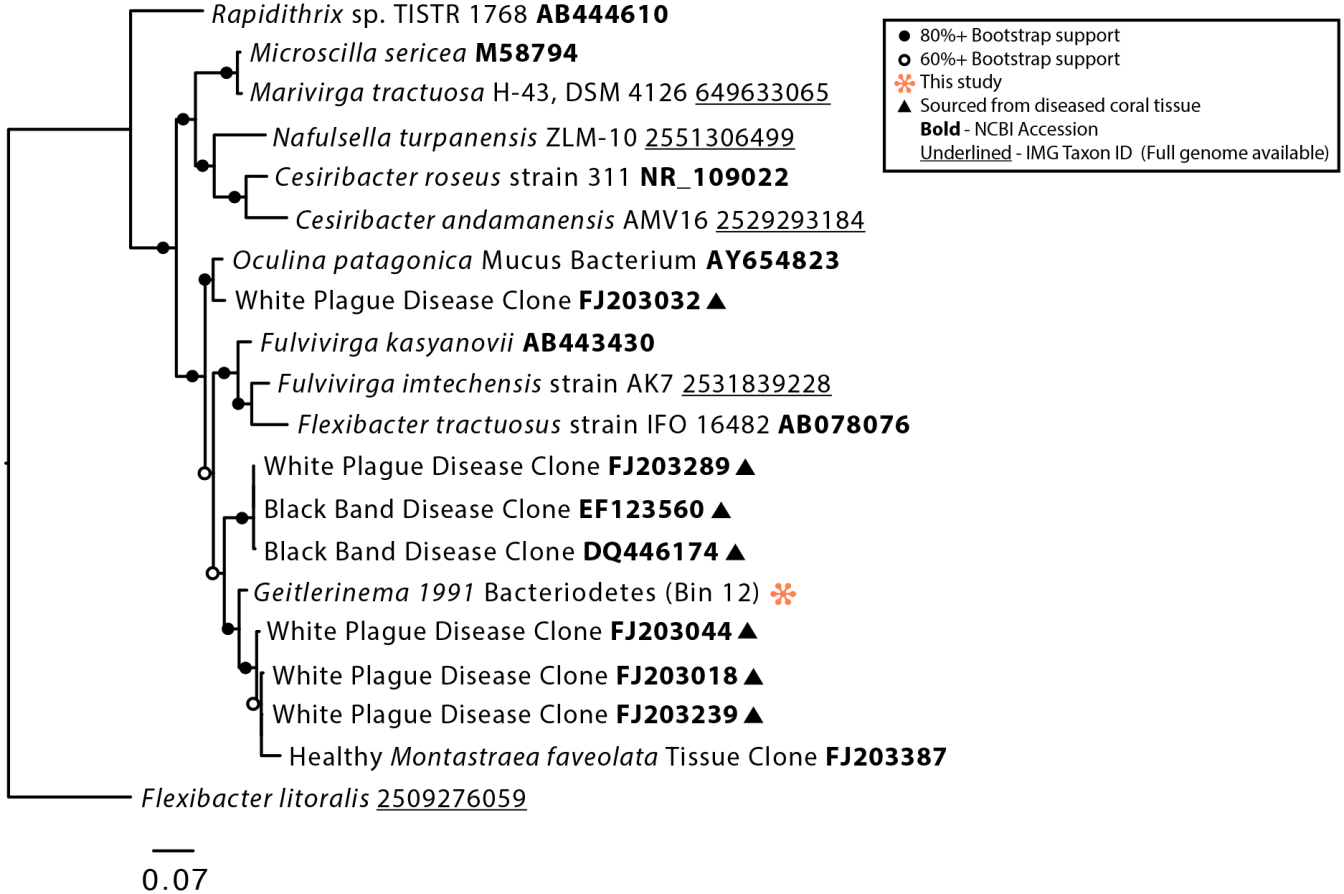
Phylogenetic tree of the 16S rRNA gene of Bin 12 and selected Bacteroidetes. Bootstrap values are the result of 5000 iterations.

**Fig 4:**
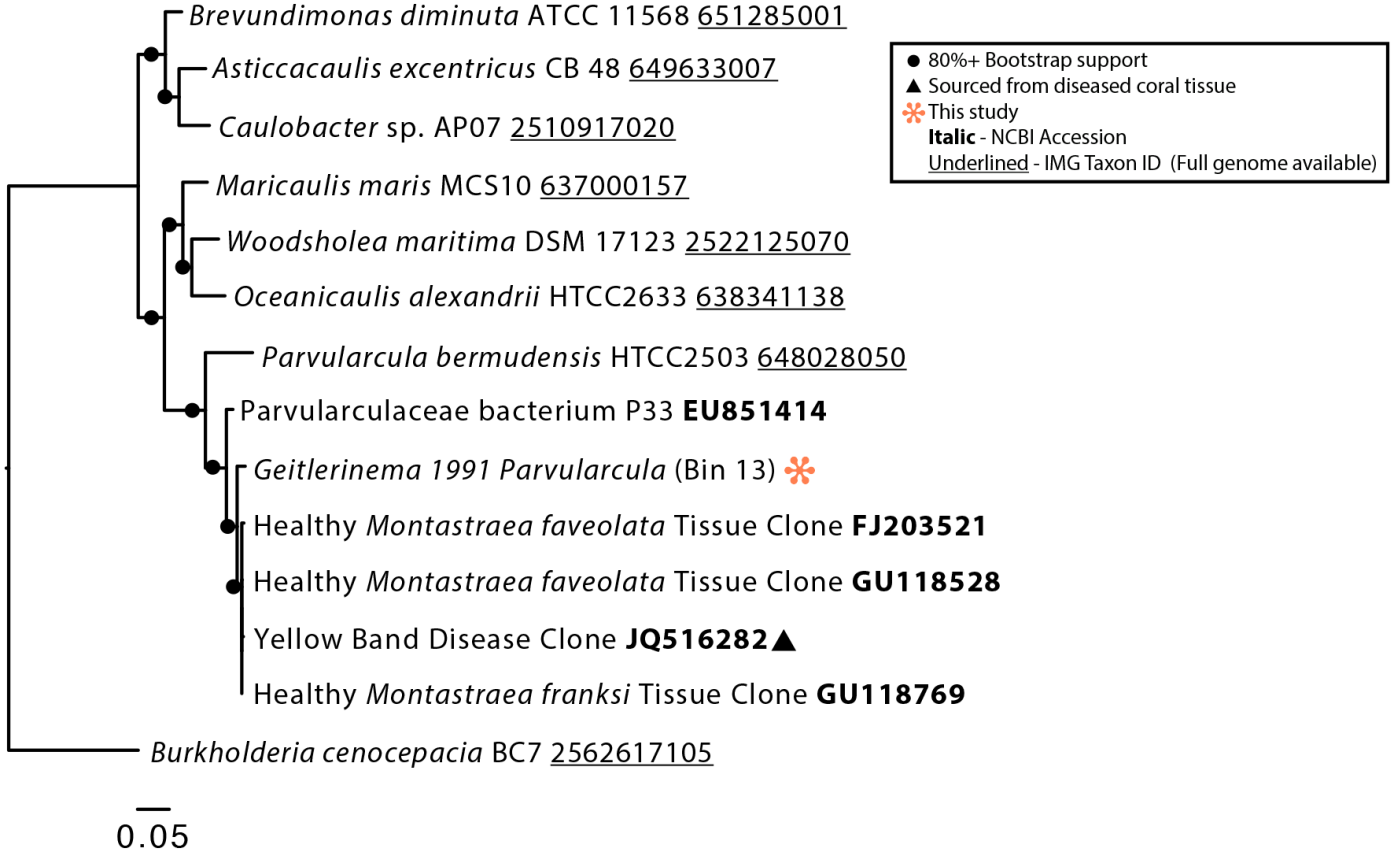
Phylogenetic tree of the 16S rRNA gene of Bin 13 and selected Alphaproteobacteria. Bootstrap values are the result of 5000 iterations.

The bacterial genomes recovered from the *Geitlerinema* BBD 1991 culture are closely related to bacteria previously found in association with both healthy and diseased corals [27, 28, 29, 50, 51, 52, 53, 54]. The 16S rRNA gene sequence from Bin 12 (Bacteroidetes; Flammeovirgaceae) clustered with sequences from the tissue of coral with the disease white plague (Fig 3). Bacteriodetes are commonly found with BBD and have been proposed to be contributors to the disease's pathogenicity [27, 28, 26]. Bin 13 was identified as an alphaproteobacterium that has close phylogenetic affiliation with sequences retrieved from both healthy corals and those afflicted with yellow band disease [54, 52, 53] (Fig 4). Below we first describe the genomic content of *Geitlerinema* sp. BBD 1991, then present highlights from the genomes of the associated bacteria.

### Photosynthesis and sulfide detoxification

As a cyanobacterium, oxygenic photosynthesis is the main energy yielding metabolism of *Geitlerinema* sp. BBD 1991. The complete sets of genes encoding photosystem I (PSI) and photosystem II (PSII) are present in the genome. Genes for the biosynthesis of chlorophyll *a*, phycobiliproteins (allophycocyanin, phycoerthrin, and phycocyanin), and red and orange carotenoid-binding proteins were also uncovered, reflecting the cyanobacteria’s ability to harvest a wide spectrum of light (S1 Table).

**Fig 5:**
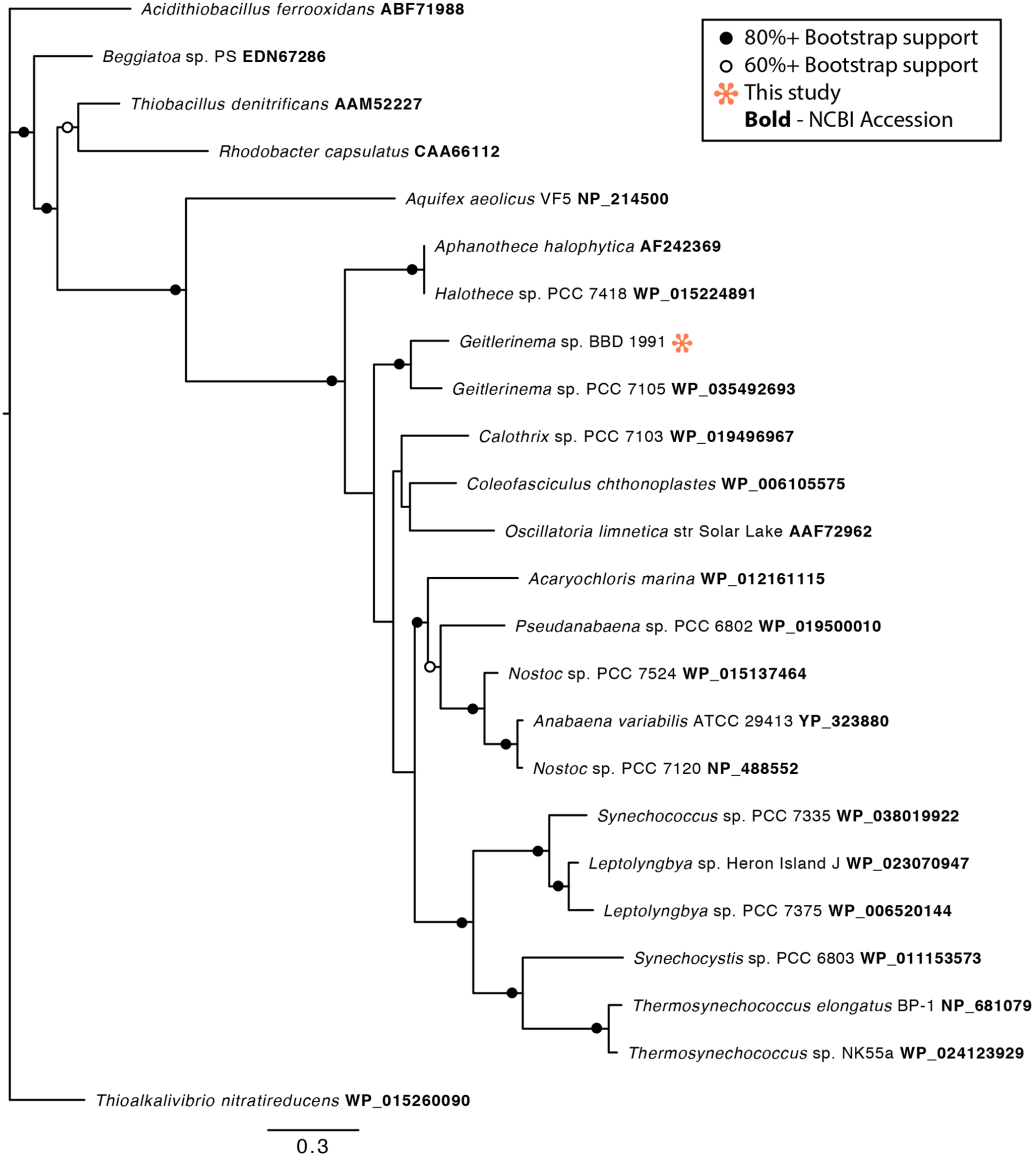
Phylogenetic tree of the predicted SQR protein sequences of *Geitlerinema* sp. BBD 1991 and selected Cyanobacteria and other Bacteria. Bootstrap values are the result of 1000 iterations.

**Fig 6:**
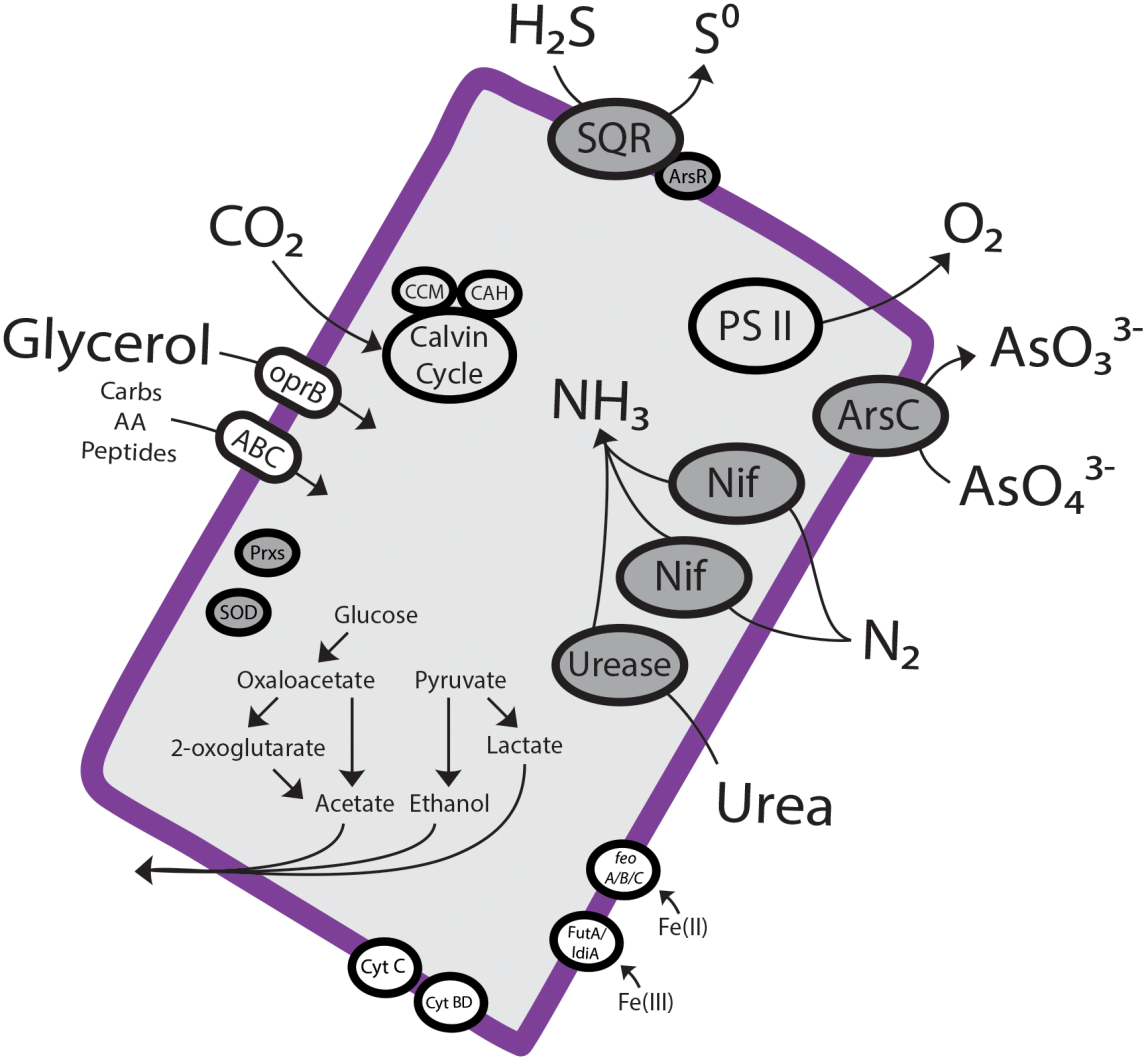
An overview of metabolism and biogeochemical cycling of *Geitlerinema* sp. BBD 1991.

The sulfide-rich environment of BBD potentially presents a challenge to *Geitlerinema* sp. BBD 1991 because in most cyanobacteria oxygenic photosynthesis is inhibited by sulfide via blockage of photosystem II [55]. Whereas some sulfide-adapted cyanobacteria can counteract this toxicity by using sulfide as an electron donor for anoxygenic photosynthesis [56], *Geitlerinema* sp. BBD 1991 does not have this capability, but rather conducts sulfide-tolerant oxygenic photosynthesis [35], a rare trait among cyanobacteria. Tolerance to sulfide by BBD cyanobacteria is crucial since the development of a population of sulfate reducing bacteria is required for the initiation of BBD [57]. The *Geitlerinema* sp. BBD 1991 genome contains at least one gene for sulfide quinone oxidoreductase (*sqr*), which has been implicated in sulfide oxidation for anoxygenic photosynthesis and also sulfide detoxification in cyanobacteria (S1 Table) [58, 59]. The *sqr* sequence in *Geitlerinema* sp. BBD 1991 clusters phylogenetically near other cyanobacteria that have been previously studied with respect to sulfide, including *Geitlerinema* sp. PCC 9228 (formerly *Oscillatoria limnetica*) [59], *Aphanothece halophytica* [59] and *Coleofasciculus chtonoplastes* (previously *Microcoleus chtonoplastes)* [55, 60, 61] (Fig 5). The lack of anoxygenic photosynthesis by *Geitlerinema* sp. BBD 1991 indicates that the *sqr* gene is likely involved in sulfide detoxification. The product of sulfide oxidation by SQR is elemental sulfur [56], which may serve as a substrate for metabolism by other BBD community members (Fig 6; see below).

**Fig 7:**
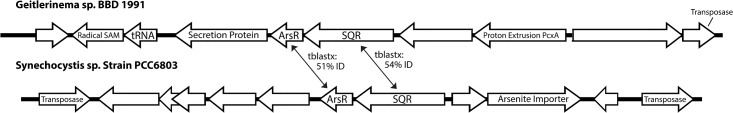
Genetic organization of the *sqr* operon of *Geitlerienema* sp. BBD 1991 (top) and *Synechocystis* sp. Strain PCC6803 (bottom; [62]). Genes denoted SQR and ArsR are thought to be involved in sulfide detoxification and arsenic resistance, respectively.

The *sqr* of *Geitlerinema* sp. BBD 1991 is organized within an apparent operon that shares characteristics with that of *Synechocystis* sp. strain PCC6803 (Fig 7), in which sulfide and arsenic detoxification have been linked. Downstream of the *sqr* gene in both *Synechocystis* sp. strain PCC6803 and *Geitlerinema* sp. BBD 1991 there is a gene encoding an ArsR-type repressor normally associated with arsenic resistance [62]. In *Synechocystis* sp. strain PCC6803, this operon is co-regulated by both arsenite and sulfide via ArsR [62]. Upstream of each respective *sqr* there is an IS*4*-like transposase, implying that this operon is a transposable element and suggesting the potential for mobility of these genes [62]. The potential role of arsenic cycling in the BBD community, if any, is unclear.

Genes encoding the entire Calvin-Benson-Bassham cycle pathway are present in the *Geitlerinema* sp. BBD 1991 genome as well as genes for CO_2_ concentrating mechanisms. The latter facilitate carbon fixation during photosynthesis by concentrating CO_2_ for rubisco and carbonic anhydrases [63], indicating mechanisms for acquisition and fixation of CO_2_ at low concentrations. Mechanisms for storage and subsequent utilization of carbon were also found in the form of genes for glycogen synthase and carbohydrate branching (1) and debranching (3) enzymes (S1 Table).

### Heterotrophy and fermentation

As mentioned above, at night the BBD band becomes fully anoxic [4] which precludes BBD community members from performing aerobic respiration, the common mode of dark energy metabolism by cyanobacteria. It has been shown that some cyanobacteria are capable of fermentation under such conditions [64]. Enhanced survival of *Geitlerinema* sp. BBD 1991 in darkness by use of exogenous organic carbon sources (both sugars and amino acids) has been demonstrated previously, but the metabolic pathway was unclear [34].

The *Geitlerinema* sp. BBD 1991 genome contains genes encoding mixed-acid fermentation (S1 Table), a pathway that may utilize (as well as generate) exogenous carbon compounds. Indeed, numerous genes for uptake of exogenous carbon were found in the form of ABC-type carbohydrate, peptide, and amino acid transporters, oprB porins, and TRAP-type C4-dicarboxylate transporters. Although the ability to utilize exogenous carbohydrates for fermentation is rare among cyanobacteria, it potentially provides a large selective advantage [64]. This is especially true in the BBD community where lysed coral tissue and coral surface mucopolysaccharide provide a plentitude of exogenous carbohydrates [5].

Glycerol is also a potential carbon source for *Geitlerinema* sp. BBD 1991 as indicated in the genome. Genes for three key enzymes of glycerol metabolism are present: glycerol kinase, glycerol-3-phosphate dehydrogenase, and triosephosphate isomerase. Although no genes annotated as glycerol uptake facilitators were found, several oprB porins present could serve this function. Use of glycerol by *Geitlerinema* sp. BBD 1991 could be advantageous because the coral’s symbiotic dinoflagellates release significant amounts of exogenous glycerol [65, 66, 67].

### Respiratory metabolism

All of the genes required for glycolysis, the citric acid cycle, and the pentose phosphate cycle are present in the *Geitlerinema* sp. BBD 1991 genome as well as genes for two different electron transport chain terminal oxidases, cytochrome *c* oxidase (subunits I, II, and III) and cytochrome *bd* plastoquinol oxidase (subunits 1 and 2) (S1 Table). Cytochrome *c* oxidase can be found in nearly all currently accessible cyanobacteria genomes, while the cytochrome *bd* plastoquinol oxidase is not as common. Cytochrome *bd* plastoquinol oxidase has a high affinity for oxygen and thus would be critical for respiration during the low O_2_ conditions in the black band environment during dusk and dawn [68].

### Nitrogen acquisition

Analysis of the *Geitlerinema* sp. BBD 1991 genome revealed multiple pathways for acquisition of nitrogen, including two operons for nitrogen fixation, genes for urea uptake and degradation, and genes encoding transporters for nitrate and ammonia (S1 Table). Such an array of nitrogen acquisition pathways may be useful for the variety of different regimes of nitrogen expected to be present within the complex BBD community. The breakdown of coral tissue by BBD would release large amounts of ammonia by deamination. On the other hand, denitrification in the band (see below) could result in limited availability of fixed nitrogen.

Two different operons for nitrogenase (*nifHDK*) found in the *Geitlerinema* sp. BBD genome suggest the potential for nitrogen fixation. Associated with both *nif* operons were genes involved in regulation of *nifB*, *nifS*, *nifU*, *nifV*, *nifE*, *nifN*, and *nifX*. *NifZ*, *nifT*, and *nifW* were adjacent to one *nif* operon, while *fdxN* was present near the other (S1 Table). Nitrogen fixation activity was not detected in previous laboratory experiments using the *Geitlerinema* BBD 1991 culture [35]. It is possible that the conditions required for nitrogen fixation are unknown, or that the *nif* genes are a relic of prior nitrogen fixing capability that has since been lost.

Hydrogenase genes, which are often closely coupled to nitrogen fixation, were also found in the *Geitlerinema* sp. BBD 1991 genome. Hydrogenases may serve two roles related to nitrogen fixation: direct consumption of H_2_, which would compete with nitrogenase activity [69], and removal of O_2_, which inhibits nitrogenase [70]. N_2_-fixing cyanobacteria often have two types of hydrogenases, uptake hydrogenase (Hup), and bidirectional hydrogenase (Hox) that can both produce and consume hydrogen [71]. The *Geitlerinema* sp. BBD 1991 genome has five bidirectional hydrogenase genes (*hoxEF*, *hoxUY*, and *hoxH*) (S1 Table) but no uptake hydrogenase. The uptake hydrogenase HupSL has been reported to be present in nearly all known N_2_-fixing cyanobacteria [69], yet our search of cyanobacterial genomes available on the JGI Integrated Microbial Genomes website (97 total at the time) showed that it is not uncommon for cyanobacteria to contain *nifHDK* genes but not *hupSL*. Of 22 *nifHDK* containing cyanobacterial genomes, only 14 had both *hupS* and *hupL*; an additional genome contained only *hupL*. It has been demonstrated that one (*Chroococcidiopsis thermalis* PCC 7203) of seven cyanobacteria that have *nifHDK* but not *hupSL* is capable of fixing nitrogen at low O_2_ concentrations (0.3–0.5%) in gas phase [69]. This could explain why neither *Geitlerinema* sp. BBD nor freshly collected BBD mat samples were able to perform nitrogen fixation when incubated in the complete absence of O_2_ [35].

### Iron cycling

Genomic signatures for iron uptake in *Geitlerinema* sp. BBD 1991 are present in the form of genes for a FutA/IdiA-based ABC transporter system together with *feoA/B/C* genes that encode and regulate putative Fe(II) transporters (S1 Table). The *feo* gene complex is present in coastal, but not open ocean, strains of *Synechococcus* [72], likely because of the greater abundance of Fe(II) in the coastal ocean due to photochemical reactions with organic matter [73]. Sunlit coral reef environments most likely exhibit similar processes.

### Secondary metabolites

**Fig 8:**
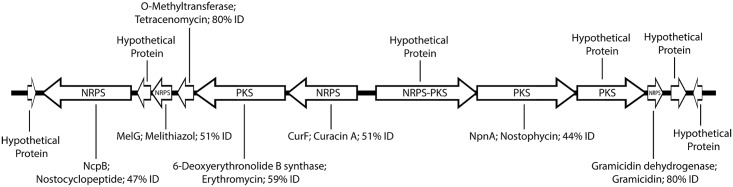
NRPS gene cluster of *Geitlerienema* sp. BBD 1991. Labels within arrows denote gene annotations, whereas those outside of the arrows show the closest TBLASTN match to a biosynthetic gene with a known product. Products and the percent identity between TBLASTN hits and query are listed next to the gene name. Arrows indicate orientation of genes.

Two uncharacterized gene clusters of nonribosomal peptide synthetases (NRPS) and two bacteriocin biosynthesis pathways were found in the *Geitlerinema* sp. BBD 1991 genome. One of the NRPS biosynthesis gene clusters is of particular interest due to its similarity to cyanotoxins (S1 Table). This NRPS gene synthesis cluster spans 34-kb in length and has NRPS, PKS (polyketide synthase), and NRPS-PKS genes (Fig 8). It features divergent operons that are oriented and transcribed in opposite directions, similar to the arrangement of the microcystin operons *mcyABC* and *mcyDEFGHIJ* in *Microcystis aeruginosa* PCC7806 [74]. Despite these similarities, this biosynthetic operon is sufficiently novel that the nature of the natural product produced is unclear.

Surprisingly absent was an operon for biosynthesis of the cyanotoxin microcystin (*mcyABCDEFG*). Microcystin is thought to be one of the key factors contributing to BBD pathogenicity as it is known to induce oxidative DNA damage [75], inhibit protein synthesis, disrupt membrane integrity and conduction [76], and induce apoptosis [77]. As mentioned above, microcystin was previously detected using analytical methods in numerous strains of Caribbean BBD cyanobacteria, including *Geitlerinema* sp. BBD 1991 [23, 24, 25]. It is possible that the *mcy* operon has been lost by *Geitlerinema* sp. BBD 1991 (in culture since 1991). We did find remnants of two genes that flank the *mcy* operon, *uma1* and *dnaN* (S1 Table). These genes are present in all known microcystin producing strains of *Microcystis* [78].

### Reactive oxygen species (ROS)

Functional genes associated with detoxification of reactive oxygen species (ROS), including superoxide dismutase and at least three peroxiredoxin genes, are present in the *Geitlerinema* sp. BBD 1991 genome (S1 Table). ROS are also commonly produced in biological systems for signaling processes and as byproducts of aerobic metabolism and photosynthesis. A buildup of ROS can result in oxidative stress, causing damage to cell proteins, lipids, and DNA. Corals produce ROS as a defense mechanism against infection [79] and oxidative stress caused by ROS has been implicated in coral bleaching [80]. Since BBD becomes supersaturated (500%) with O_2_ in midday [4], a mechanism to protect against ROS damage would be of obvious benefit in the BBD environment.

### Genomic insights into the potential ecological significance of BBD-associated bacteria

**Fig 9:**
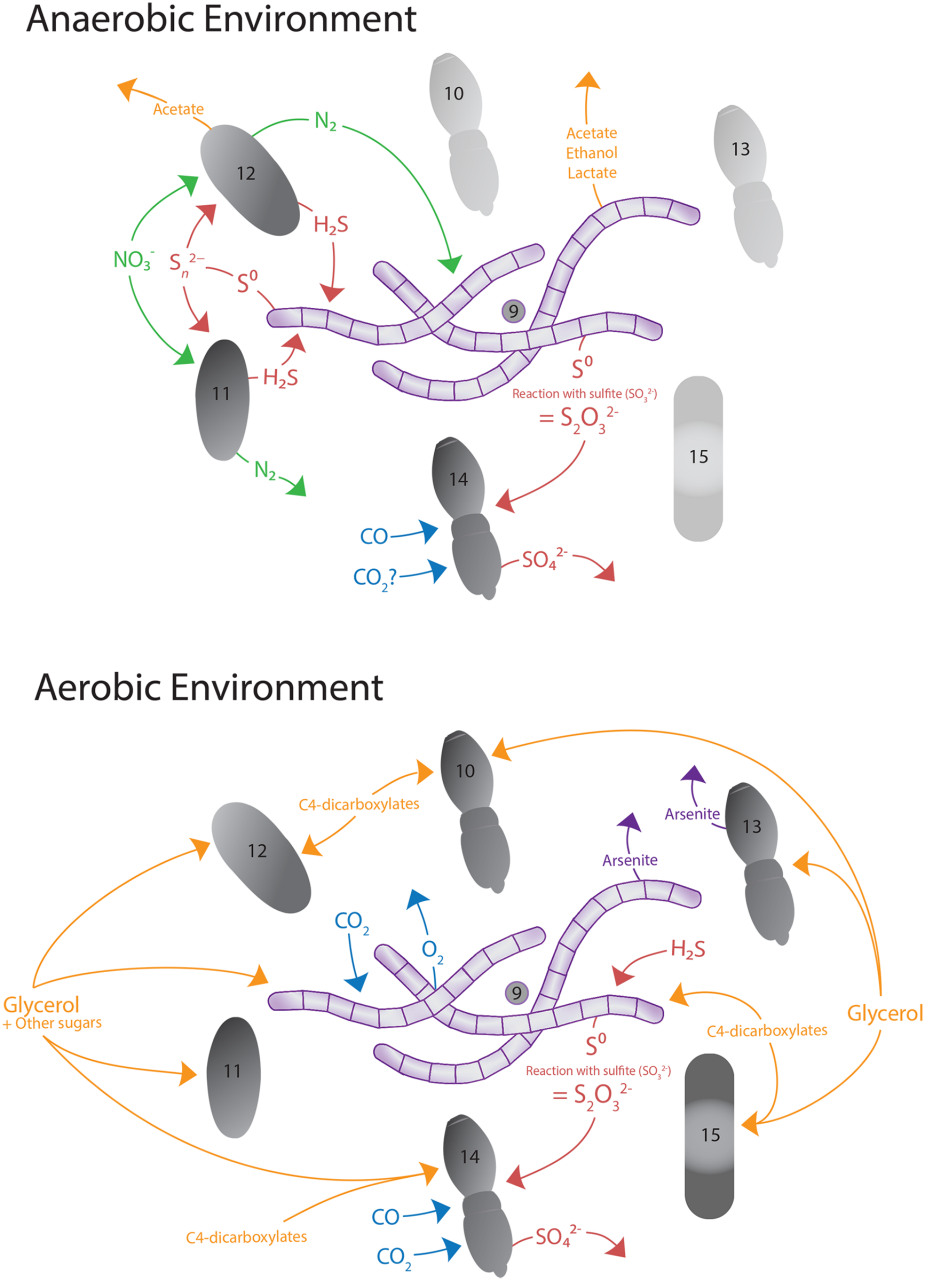
Proposed nutrient and carbon cycling between organisms present in the *Geitlerinema* sp. BBD 1991 culture in anaerobic and aerobic conditions. Numbers indicate bin numbers shown in Table 1. Symbols representing bacterial cells courtesy of the Integration and Application Network, University of Maryland Center for Environmental Science (ian.umces.edu/symbols/).

Given the close phylogenetic relationships between bacteria in the *Geitlerinema* BBD 1991 culture and sequences retrieved from culture independent studies of bacteria associated with *in situ* corals, we analyzed the genomic bins of these co-cultured bacteria to identify potential metabolic interactions (Fig 9). Of particular interest are genes involved in sulfur cycling, especially processes that produce and consume sulfide, one of the key determinants of BBD virulence. Bin 12 (Bacteroidetes; Flammeovirgaceae) and Bin 11 (Planctomycetes; Planctomycetaceae) have genes for polysulfide reductases, which convert polysulfide to hydrogen sulfide and may be coupled to energy conservation via the electron transport chain [81] (S1 Table). Polysulfide targeted by these reductases may have been generated spontaneously from the oxidation of both elemental sulfur and sulfide [82]. Elemental sulfur would be expected to be present in the form of extracellular sulfur globules via SQR mediated sulfide oxidation [56], and intracellular sulfur granules have been observed microscopically in *Beggiatoa* filaments present in freshly collected BBD samples [5]. Close relatives of Bin 12 have previously been observed to be associated with BBD (Fig 3), suggesting that a polysulfide reductase mechanism may be relevant to the production of sulfide *in situ*.

Bin 14 (Alphaproteobacteria; Rhodospirillales) contains the full *sox* pathway (*soxRSV-XYZ-ABCD-EFH*; S1 Table) allowing for lithotrophic oxidation of thiosulfate [83]. A putative *soxW* is also present (55% amino acid identity to *soxW* from Stappia Stellulata; WP_037546634). Bin 13 (Alphaproteobacteria; Parvularculaceae) contained evidence of aerobic anoxygenic phototrophy in the form of *puf* genes coding for the *pufA* subunit of the light-harvesting complex and reaction centre complex (*pufM* and *pufL)* [84]. Although aerobic anoxygenic photosynthesis is a common trait found in marine alphaproteobacteria, this is the first report of it in the family Parvularculaceae, and the first indication that anoxygenic photosynthesis is operating in BBD.

Consistent with metabolic expectations of (co-cultured) epibionts of cyanobacteria, evidence for organoheterotrophy was observed in all of the genomic bins. Bin 10 (Alphaproteobacteria; Hyphomonadaceae) has genes predicted to encode uptake of organic carbon, including oligopeptide ABC-transporters, an ABC-type amino acid transporter, and a TRAP-type transporter. Bin 12 (Bacteroidetes; Flammeovirgaceae) contained genes for pyruvate fermentation to acetate and ABC-type sugar and TRAP-type transporters. Interestingly, Bin 12 and Bin 11 (Plantomycetes; Planctomycetaceae) also contain pathways for denitrification, including nitrate reductase, nitrite reductase, nitric oxide reductase (Bin 12 only), and nitrous oxide reductase (Bin 12 only) (S1 Table). Bin 14 (Alphaproteobacteria; Parvularculaceae) also contained genes for TRAP-type C4-dicarboxylate transporters, ABC-type sugar and amino acid transporters.

As in the *Geitlerinema* sp. BBD 1991 genome, we found evidence for glycerol metabolism in the genome bins of all associated bacteria. All bins have genes for glycerol degradation (S1 Table), and bins 10, 12, 14, and 15 also had genes explicitly for glycerol uptake. The ubiquitous ability of *Geitlerinema* sp. BBD 1991 and its co-cultured bacteria to use glycerol highlights the importance of glycerol as a carbon source for coral-associated bacteria. Another theme consistent across the genomes analyzed was the presence of genes encoding ROS defense (discussed above), with various combinations of superoxide dismutase and peroxiredoxin genes in each bin (S1 Table).

We recovered a number of genes for production of and resistance to antibiotics, which may influence the structure and succession of microbial communities [85] including those associated with coral [37] (S1 Table). Bin 12 has genes predicted to encode production of both stringomycin and gramicidin (S1 Table). Bins 10, 12, 13, 14, and 15 have genes for MarR transcriptional regulators that regulate a wide variety of biological functions, in particular multiple antibiotic resistance as well as other mechanisms of defense. All bins had genes encoding betalactamase or 4-hydroxy-3-methylbut-2-enyl diphosphate reductase (penicillin tolerance protein), further indicating antibiotic resistance (S1 Table). Overall, these results suggest that the BBD community is a rich source of secondary metabolites and that mechanisms of antibiotic and/or toxin resistance are essential for survival in this environment.

## Conclusions

The genome sequence of *Geitlerinema* sp. BBD 1991 points to a versatile lifestyle with diverse mechanisms of nutrient acquisition and energy metabolism. There is genetic potential for uptake of a wide range of nitrogen compounds, from N_2_ to urea and ammonia, and for both reduced and oxidized forms of iron. The sulfide-resistant photosynthetic function is underpinned by sulfide quinone oxidoreductase. Genes for both photoautotrophic and heterotrophic metabolism are present. Also notable are genes that reflect diverse mechanisms for defense against potential insults from other community members, including antibiotics and reactive oxygen species.

The metagenome of the *Geitlerinema* BBD 1991 culture provides novel insight into metabolisms and eco-physiologies of microorganisms that are associated with BBD. In particular, the metagenome highlights the potential importance of cycling of sulfur intermediates, such as conversion of polysulfide and thiosulfate to sulfate and sulfide. These products could then be used as electron acceptors and donors in anaerobic respiration and lithotrophy, respectively. The metagenome also points to utilization of diverse organic carbon substrates. Additional metabolic processes such as denitrification of nitrate to N_2_, scavenging of reactive oxygen species, and production of natural products are all likely to shape environmental conditions and microbial community structure of the BBD mat.

Overall, the genetically encoded traits of the cyanobacterium *Geitlerinema* sp. BBD 1991 and its associated bacteria are consistent with adaptations to a dynamic environment with rapidly changing redox conditions and extensive biological interactions. Currently, a metagenomic analysis of the BBD cyanobacterium *Roseofilum reptotaenium* is underway, which will provide an interesting comparison of the metabolic potential between this globally distributed BBD cyanobacterium and the Caribbean *Geitlerinema* sp. BBD 1991.

## Supporting Information

S1 Table Table of genes from the culture *Geitlerinema* BBD 1991. Name, respective bin (organism), and IMG ID are included for each gene. Additional genes of interest not discussed in this article are included.
(XLSX)
